# A Targeted-Covalent Inhibitor of 17β-HSD1 Blocks Two Estrogen-Biosynthesis Pathways: In Vitro (Metabolism) and In Vivo (Xenograft) Studies in T-47D Breast Cancer Models

**DOI:** 10.3390/cancers13081841

**Published:** 2021-04-13

**Authors:** Donald Poirier, Jenny Roy, René Maltais

**Affiliations:** 1 Laboratory of Medicinal Chemistry, Endocrinology and Nephrology Unit, CHU de Québec—Research Center, Québec, QC G1V 4G2, Canada; jenny.roy@crchudequebec.ulaval.ca (J.R.); rene.maltais@crchudequebec.ulaval.ca (R.M.); 2 Department of Molecular Medicine, Faculty of Medicine, Université Laval, Québec, QC G1V 0A6, Canada

**Keywords:** breast cancer, estrogen, hormone, steroid, inhibitor, DHEA, estrone

## Abstract

**Simple Summary:**

17β-Hydroxysteroid dehydrogenase type 1 (17β-HSD1) is responsible for the production of estrogens estradiol (E2) and 5-androsten-3β,17β-diol (5-diol). This enzyme is therefore a target of choice for the treatment of estrogen-dependent diseases such as breast cancer and endometriosis, by blocking estrogen biosynthesis. After we developed the first irreversible and non-estrogenic 17β-HSD1 inhibitor, a molecule named PBRM, our goal was to demonstrate its therapeutic potential. PBRM was able to block the formation of E2 and 5-diol in T-47D human breast cancer cells. When given orally to mice, PBRM was also able to block the tumor growth without any observed toxic effects. Thanks to its irreversible type of inhibition, PBRM retained its anti-tumor growth effect, even after reducing its frequency of administration to only once a week, a clear advantage over reversible inhibitors. These results strongly support the use of PBRM as a new approach in the treatment of breast cancer.

**Abstract:**

17β-Hydroxysteroid dehydrogenase type 1 (17β-HSD1) plays an important role in estrogen-dependent breast tumor growth. In addition to being involved in the production of estradiol (E2), the most potent estrogen in women, 17β-HSD1 is also responsible for the production of 5-androsten-3β,17β-diol (5-diol), a weaker estrogen than E2, but whose importance increases after menopause. 17β-HSD1 is therefore a target of choice for the treatment of estrogen-dependent diseases such as breast cancer and endometriosis. After we developed the first targeted-covalent (irreversible) and non-estrogenic inhibitor of 17β-HSD1, a molecule named PBRM, our goal was to demonstrate its therapeutic potential. Enzymatic assays demonstrated that estrone (E1) and dehydroepiandrosterone (DHEA) were transformed into E2 and 5-diol in T-47D human breast cancer cells, and that PBRM was able to block these transformations. Thereafter, we tested PBRM in a mouse tumor model (cell-derived T-47D xenografts). After treatment of ovariectomized (OVX) mice receiving E1 or DHEA, PBRM given orally was able to reduce the tumor growth at the control (OVX) level without any observed toxic effects. Thanks to its irreversible type of inhibition, PBRM retained its anti-tumor growth effect, even after reducing its frequency of administration to only once a week, a clear advantage over reversible inhibitors.

## 1. Introduction

Breast cancer is the most common cancer in women and the second leading cause of cancer death [1]. Approximately 70% of breast cancers are estrogen-dependent, which means that they are likely to respond positively to treatment targeting either the action or formation of estrogens. To block the action of estrogens on the estrogen receptor alpha (ERα), several families of antagonists (pure antiestrogens and selective estrogen receptor modulators) have been developed [2,3,4,5]. Although these molecules are effective and commonly used in clinical practice, some patients develop resistance [6], hence the interest in working on other therapeutic agents such as enzyme inhibitors that would reduce the concentration of estrogens.

**Figure 1:**
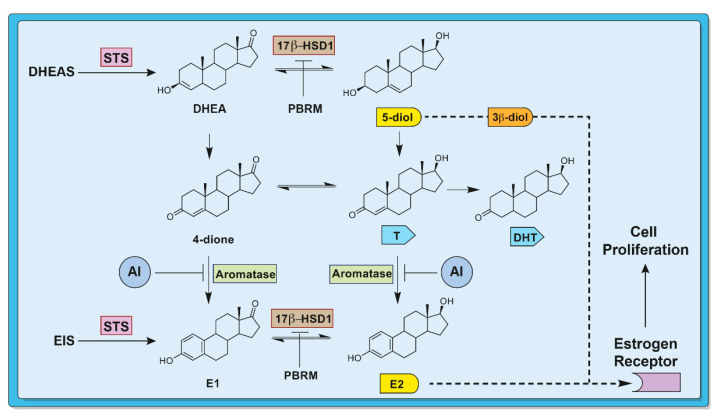
Involvement of three enzymes (STS, 17β-HSD1 and aromatase) in the formation of estrogens (5-diol and E2) in breast cancer cells and tumors. The formation of 5-diol is not prevented by an aromatase inhibitor (AI), unlike with the use of a 17β-HSD1 inhibitor such as PBRM.

Several enzymes are involved in the synthesis of estrogens [7], but aromatase (CYP19A1; EC 1.14.14.1), steroid sulfatase (STS; EC 3.1.6.2) and 17β-hydroxysteroid dehydrogenase type 1 (17β-HSD1; EC 1.1.1.51) have been targeted for the development of inhibitors for therapeutic purposes (Figure 1) [8,9,10,11,12]. Of these enzymes, aromatase is the best known, since it is involved in the aromatization of the A-ring of C19-steroids to obtain the C18-steroids estrone (E1) and estradiol (E2) [13]. Until now, aromatase is the only one of these three enzymes whose development of inhibitors has led to marketed drugs to treat breast cancer, namely anastrozole, letrozole and exemestane [14]. The development of STS inhibitors is more recent but has also given rise to a great deal of work [15,16,17,18,19,20]. This enzyme is responsible for the hydrolysis of sulfated hydroxysteroids to free hydroxysteroids, the form generally active on a receptor or which can serve as precursor for estrogen biosynthesis. Only one of these STS inhibitors (Irosustat) has reached clinical trials (phase II) showing clinical benefits when combined with an aromatase inhibitor, but larger studies are required before to be translating this to a marketed drug to treat breast cancer [21,22,23]. The third enzyme, 17β-HSD1, also has an important role in estrogen synthesis and tumor growth [24,25,26,27,28]. In addition to being directly involved in the production of E2, the most potent estrogen in women, from E1, this enzyme is also responsible for the production of 5-androstene-3β,17β-diol (5-diol) from dehydroepiandrosterone (DHEA), a less potent estrogen than E2 [29,30], but a compound whose production increases after menopause (Figure 1). 17β-HSD1 is therefore a target of choice for the treatment of breast cancer because its inhibition would make it possible to obtain optimal blocking of estrogen biosynthesis (E2 and 5-diol) and of estrogen-dependent breast cancer cell proliferation.

**Figure 2:**
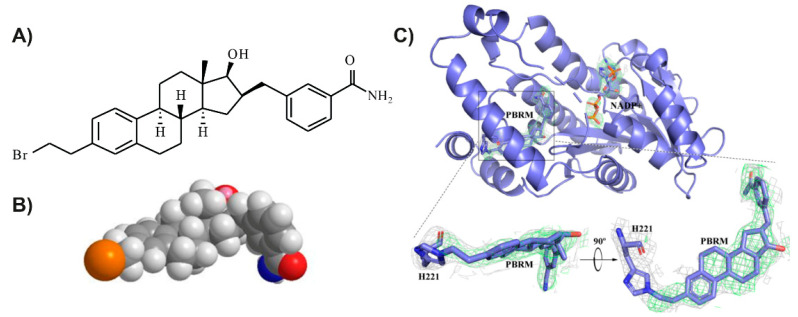
The irreversible steroidal 17β-HSD1 inhibitor PBRM: (**A**) two-dimensional (2D) representation; (**B**) three-dimensional (3D) representation; and (**C**) PBRM linked covalently to 17β-HSD1 [45].

The development of 17β-HSD1 inhibitors has given rise to several studies [31,32,33,34,35,36,37,38], mainly at the beginning of the 21st century, but this research has not yet produced a drug to treat breast cancer [39]. However, a 17β-HSD1 inhibitor is currently undergoing phase 1 clinical studies for use in treating endometriosis [40]. This inhibitor, as well as most inhibitors developed, are of the reversible type, steroidal and nonsteroidal [31,32,33,34,35,36,37,38]. Our research group has developed a targeted-covalent (irreversible) inhibitor of 17β-HSD1 (Figure 2), which has shown promising results in cell assays and in mice [41,42,43,44,45]. Using this inhibitor (PBRM), our goal is now to demonstrate its potential for the treatment of estrogen-dependent breast tumors stimulated by E1 as well as by DHEA. By using cell-derived T-47D xenografts, we also hope to demonstrate the role of 5-diol as an alternative source of estrogen in the stimulation of hormone-dependent breast cancer tumors.

## 2. Results

### 2.1. DHEA as Precursor of Estrogenic Effects

The estrogenic hormone E2 is produced from DHEA using the aromatase and 17β-HSD1 pathway via the formation of 4-androstene-3,17-dione (4-dione) (Figure 1). An alternative route which would involve the transformation of 4-dione into testosterone (T) and its aromatization into E2 has also been proposed, but, 4-dione being a much better substrate for aromatase than T, this route seems unlikely [46,47]. However, it has been suggested that 5-diol produced from DHEA could serve as an alternative source of estrogens after the ovaries stop the production of E2, at menopause [48]. Despite its 19-carbon steroid nucleus, unlike E2 (18 carbons), 5-diol has affinity for ERα (RBA = 6% and 100% for 5-diol and E2, respectively) [30] and it would be able to produce an estrogenic effect, such as stimulating the proliferation of ERα-positive cells [29,48,49]. To take into account this second pathway, which could be responsible for the production of estrogens and consequently the stimulation of breast cancer tumors, we tested DHEA as a source of estrogen in human T-47D cells, which are known for their high content of 17β-HSD1 [50], as well as in mouse xenograft tumor models.

In preliminary experiments, different numbers of human breast cancer T-47D cells (3000/6000/12,000 and 24,000) were incubated for different periods (1, 2, 3, 6 and 8 days) in the presence of [^14^C]-DHEA and [^14^C]-4-dione (Figures S1–S6 and Tables S1–S7). Analysis by thin layer chromatography (TLC) of the metabolites showed the gradual disappearance of DHEA and the gradual formation of 5-diol as a function of cell number and time, while other metabolites (E1, E2, T, DHT, androsterone (ADT) and 5α-androstane-3β,17β-diol (3β-diol)) were present but in very low quantity. When we used 4-dione as a substrate, the observations were similar to those obtained with DHEA except that 5α-androstane-3,17-dione (A-dione) was the dominant metabolite instead of 5-diol. In both cases, however, the conversion rate of the substrate used was very low. From its preliminary data, we then selected optimal conditions (24,000 cells and 8 days) to study the metabolism of DHEA in the presence or not of 17β-HSD1 (PBRM) and aromatase (Letrozole; LET) inhibitors. Steroid quantification by gas chromatography tandem mass spectrometry (GC-MS/MS), a more efficient analytical method than TLC, made it possible to confirm the presence of substrate and key metabolites (Table 1).

During incubation of DHEA in T-47D cells, the main metabolite observed with 21.0% of the quantified steroids was 5-diol, which results from the transformation of DHEA by 17β-HSD1 (Table 1). 4-Dione (1.0%) was the second metabolite observed, while the potent hormones E2 (estrogen) and DHT (androgen) were observed in very low quantity. The analytical method did not allow the determination of A-dione, the product of the double bond (Δ^4,5^) reduction by 5α-reductase, but preliminary experiments using radioactive DHEA as substrate nevertheless showed a very low quantity of A-dione as metabolite. In the presence of the 17β-HSD1 inhibitor PBRM, there is a decrease of 33% of 5-diol (from 21.0% to 13.9%) and an increase of 10% of DHEA (from 77.9% to 84.7%). As expected, this increase of DHEA level is observed from the PBRM blocking action of DHEA transformation to 5-diol. In contrast, the use of aromatase inhibitor LET did not affect the concentrations of DHEA and 5-diol, while the results observed with the combination of PBRM and LET are similar to those obtained with the use of PBRM alone. In addition to showing the impact of a 17β-HSD1 inhibitor, the results of DHEA metabolization assays suggest a very weak production of E2, which could be explained by a negligible contribution of aromatase in T-47D cells. This result is in line with literature reports showing a weak production of E2 from 4-dione in these cells, despite the presence of aromatase [51,52]. For example, Ryde et al. reported that only 4 pM of E1 was produced from 4-dione with four million T-47D cells incubated for 24 h [51]. This breast cancer cell line is thus an appropriate model to measure the estrogenic effect of 5-diol.

**Figure 3:**
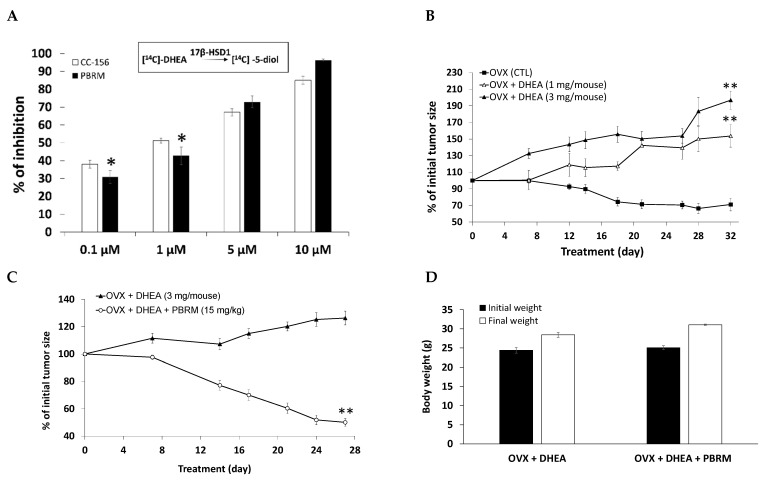
(**A**) Inhibition (%) of 5-diol formation from DHEA in human breast cancer T-47D cells. Cells (24,000/well) were incubated for eight days in the presence of the radioactive substrate ([^14^C]-DHEA) and the inhibitor PBRM (IC_50_ = 0.77 µM for PBRM and 0.52 µM for CC-156). (*) *p* < 0.05 vs. CC-156. (**B**) Effect of DHEA injected SC (6 days/week) in propylene glycol:EtOH (92:8) on breast cancer tumor growth (T-47D xenografts) in nude mice. (**) *p* < 0.01 vs. OVX (CTL) at 12, 14, 18, 21, 26, 28 and 32 days (except *p* < 0.5 at 12 and 14 days for 1 mg/kg). (**C**) Inhibition (%) of breast cancer tumor growth (T-47D xenografts) stimulated by DHEA (3 mg/mouse) injected SC (6 days/week) in nude mice. The inhibitor PBRM was solubilized in sunflower:EtOH oil (92:8) and administered PO (by gavage) six days a week. (**) *p* < 0.01 vs. OVX + DHEA at 7, 14, 17, 21, 24 and 27 days. (**D**) Body weight of mice from the protocol reported in (**C**). No significant difference between PBRM-treated group and untreated group (OVX + DHEA).

PBRM inhibits the formation of 5-diol from DHEA by T-47D cells, in a dose-dependent manner, since the inhibition gradually increased from 30% to 100% depending on the 4 concentrations tested and varying from 0.1 to 10 μM (Figure 3A). In this cell culture assay with DHEA as estrogen precursor, it was observed that the efficacy of the irreversible inhibitor PBRM (IC_50_ = 0.77 μM) is similar to that of its closely related reversible analogue CC-156 [53] showing an IC_50_ of 0.52 μM, which was also the case when E1 was used as a substrate (IC_50_ = 68 and 27 nM for PBRM and CC-156, respectively) [43].

After confirming the efficacy of PBRM to inhibit the formation of 5-diol in T-47D cells, we extended our study using an in vivo model of breast cancer, namely T-47D cell xenografts in ovariectomized (OVX) nude mice. In a preliminary study, we first determined that a dose of 3 mg/mouse/day of DHEA was preferable to 1 mg/mouse/day to promote tumor growth, over time (Figure 3B). Using this dose, we then observed that PBRM (15 mg/kg) given orally (PO, gavage) effectively reversed T-47D tumor growth (Figure 3C) to a level comparable to that of untreated OVX mice (Figure 3B). In addition, analysis of the behavior of the mice during the protocol or of their body weight (Figure 3D) did not show any sign of apparent toxicity of PBRM. The results of this first in vivo experiment using DHEA as an estrogen precursor are very encouraging, but they will still need to be confirmed by new xenograft experiments with additional groups.

### 2.2. E1 as Precursor of Estrogenic Effects

**Figure 4:**
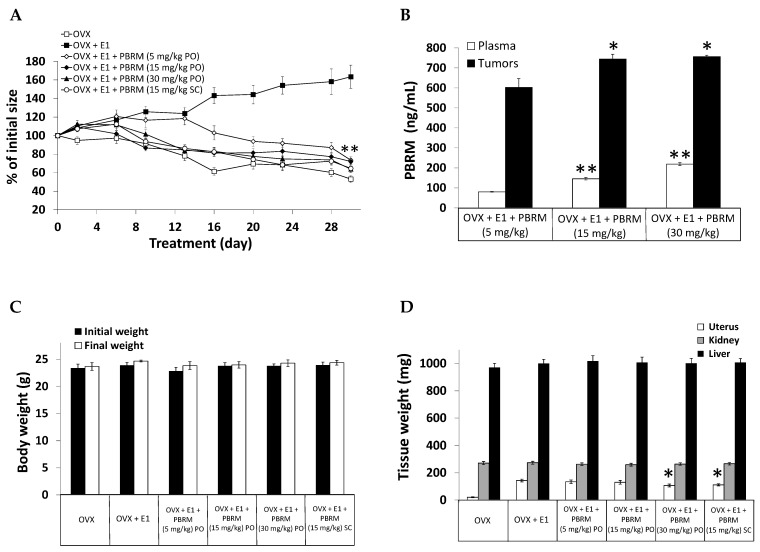
(**A**) Inhibition (%) of breast cancer tumor growth (T-47D xenografts) stimulated by E1 injected subcutaneously (SC) six days a week in nude mice. The inhibitor PBRM was solubilized in sunflower oil:EtOH (92:8) and administered PO (gavage) or SC in propylene glycol:EtOH (92:8) six days a week. (**) *p* < 0.01 vs. OVX + E1 at 16, 20, 23, 28 and 30 days. (**B**) Plasma and tumor concentrations of PBRM at the end of the protocol are reported in (**A**) (3 h after the last PBRM administration). (*) *p* < 0.05 and (**) *p* < 0.01 vs. OVX + E1 + PBRM (5 mg/kg). (**C**) Body weight of mice from the protocol reported in (**A**). No significant difference between PBRM-treated groups and untreated group (OVX + E1). (**D**) Uterus, kidney and liver weights of mice from the protocol reported in (**A**). No significant difference between PBRM-treated groups and untreated group (OVX + E1) except for uterus (*) *p* < 0.05.

The inhibitor PBRM has already shown its ability to block the formation of the most potent estrogen, E2, from the precursor E1 (IC_50_ = 68 nM) in T-47D cells [43] and a proof of concept had been carried out in a model of breast cancer tumors (T-47D xenografts in OVX mice), but at a single dose only and using the subcutaneous (SC) mode of administration [42]. Using the same model, where 17β-HSD1 activates E1 to E2, thus stimulating the growth of estrogen-dependent T-47D tumors, we reproduced the result initially obtained with the same dose (15 mg/kg) and SC mode (Figure 4A). In this experiment, we also observed the effectiveness of PBRM to block tumor growth when given orally at the same dose of 15 mg/kg in a mixture of sunflower oil:EtOH (92:8). At 30 mg/kg PO, the result is similar to those obtained at 15 mg/kg PO and SC, and no significant difference was observed at the end of the protocol. At the lower dose of 5 mg/kg PO, PBRM also reversed tumor growth, but 16 days was needed, as opposed to only nine days for the higher doses. At the end of the protocol, the blood content of PBRM 3 h after the last administration (Figure 4B) showed that the concentration of the inhibitor increased (80, 150 and 210 ng/mL) with the dose (5, 15 and 30 mg/kg, respectively), which explains the lower efficacy observed in the first days of the protocol with only 5 mg/kg PO. After 30 days of treatment (Figure 4B), the concentration of PBRM in tumors varied less (602, 745 and 757 ng/mL at 5, 15 and 30 mg/kg, respectively), but it was lower at the lower dose of 5 mg/kg.

Analysis of the mouse behavior during the 30 days of treatment and the body weight at the end of the protocol (Figure 4C) did not show any sign of apparent toxicity of PBRM. No significant weight loss/gain of liver and kidney was detected between PBRM-treated and untreated groups (Figure 4C). For the uterus, an estrogen-sensitive tissue, its weight increased from 20 (OVX) to 143 mg when treated by E1 (0.1 μg/day, SC), and, as expected, no significant weight difference was observed between the group treated with E1 and those treated with E1 and PBRM at 5 and 15 mg/kg PO (Figure 4D). In fact, unlike T-47D tumor stimulation by E1 injected SC in OVX mice, and under the control of human 17β-HSD1 (by producing E2), the stimulation of the uterus by E1 is under the control of mouse 17β-HSD1, and PBRM did not irreversibly inhibit this enzyme because of the absence of His-221 in this species [44]. However, a small difference (*p* < 0.05) appears with the groups treated with PBRM at 30 mg/kg PO and 15 mg/kg SC (107 and 111 mg) vs. the OVX + E1 group (143 mg). This effect, observed only at the two doses producing the higher blood concentrations of PBRM (Figure 4B), can however be explained by the weak reversible inhibitory activity of PBRM on mouse 17β-HSD1 [44].

### 2.3. Assessment of PBRM Frequency of Administration

**Figure 5:**
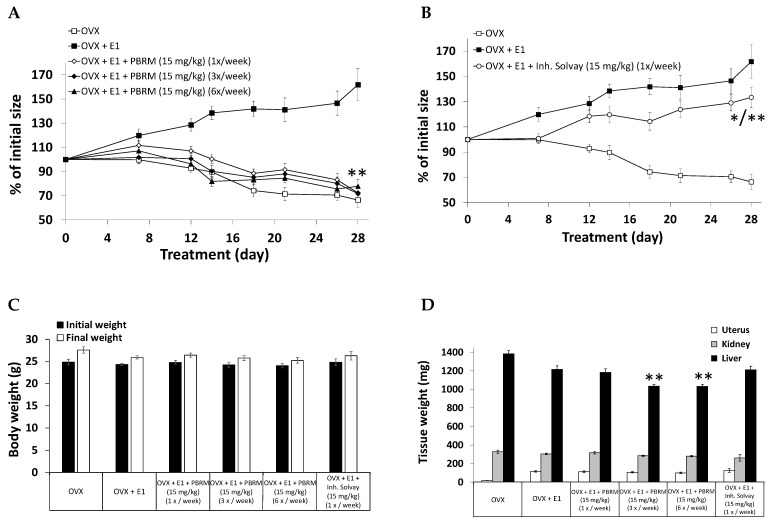
(**A**) Inhibition (%) of breast cancer tumor growth (T-47D xenografts) stimulated by E1 injected SC six days a week in nude mice. Irreversible inhibitor PBRM (15 mg/kg) was solubilized in sunflower oil:EtOH (92:8) and administered PO (gavage) at three different frequencies (1, 3 and 6 times/week). The reversible Solvay’s inhibitor (compound **21** in [57]) (15 mg/kg) was solubilized in sunflower oil:EtOH and administered PO at a frequency of 1 time/week. (**) *p* < 0.01 vs. OVX + E1 at 12, 14, 18, 21, 26 and 28 days. (**B**) Inhibition (%) of breast cancer tumor growth (T-47D xenografts): Comparison between both types (reversible and irreversible) of 17β-HSD1 inhibitors (15 mg/kg) at the lower frequency of administration (1 time/week). (*) *p* < 0.05 vs. OVX + E1 and (**) *p* < 0.01 vs. OVX at 12, 14, 18, 21, 26 and 28 days. (**C**) Body weight of mice from the protocol reported in (**A**,**B**). No significant difference between PBRM-treated groups and untreated group (OVX + E1). (**D**) Uterus, kidney, and liver weights of mice treated in the protocol reported in (**A**,**B**). No significant difference between the treated groups and untreated group (OVX + E1) except for liver (**) *p* < 0.01 vs. OVX + E1.

PBRM is an irreversible inhibitor of 17β-HSD1, which differentiates it from reversible inhibitors developed for this enzyme. Although not frequently used as a drug until recently, because of the side effects associated with the poor selectivity of first-generation irreversible inhibitors, targeted-covalent inhibitors are now attracting interest for their special properties [54,55,56], such as the possibility of reducing the frequency of administration. Using OVX nude mice as a model (T-47D xenografts) of E1-stimulated estrogen-dependent tumors (after transformation by 17β-HSD1 to E2), we observed that PBRM was active even after reducing its frequency of administration per week from 6 to 3, then finally as little as 1 (Figure 5A). Thus, at the selected dose of 15 mg/kg administered orally (PO), PBRM gradually reduced tumor growth at each frequency (1, 3 and 6 times/week) to quickly reach the level of the control group (not stimulated by E1) towards 14 days. Throughout the protocol, PBRM produced its effect more quickly with increasing frequency of administration, but this difference was not statistically significant. Indeed, a single oral administration of PBRM by week was able to completely reverse the growth of tumors stimulated by E2, generated from E1, thus demonstrating a sustained blockade of 17β-HSD1. Given once a week, a reversible 17β-HSD1 inhibitor of Solvay (compound **21** in [57]) used for comparison was not able to effectively reverse tumor growth (Figure 5B). For this reversible inhibitor, a higher dose, or an increase in the frequency of administration would therefore have been necessary, which is not needed for the irreversible inhibitor PBRM. Similar to other xenograft experiments, no significant behavior issue was observed as well as weight loss/gain of body, kidney and uterus vs. control group treated with E1 (Figure 5C,D). In fact, no apparent toxicity was detected during the 28 days of treatment.

## 3. Discussion

The estrogenic effect has long been associated with the formation of estradiol (E2), the most potent estrogenic female hormone. It is therefore not surprising that strategies to counteract the detrimental action of estrogens on the proliferation of breast cancer cells have mainly focused on blocking the estrogen receptor (antiestrogens) and subsequently on blocking the formation of E2 (inhibitors of aromatase, STS, and certain 17β-HSDs). Some rare studies have however focused on the importance of other sources of estrogens, and in particular on 5-diol, a C19-steroid that more weakly binds ERα, but whose importance is believed to become more pronounced after menopause, especially in breast tumor growth.

One of our study’s main objectives was to demonstrate the effect of 17β-HSD1 on the biosynthesis of estrogen 5-diol from DHEA in breast cancer, in addition to the blockade of E1 to E2 transformation. Among the well-recognized estrogen-sensitive ER(+) human breast cancer cells (T-47D, MCF-7 and ZR-75-1), we selected the T-47D cell line, since it expresses a very low level of aromatase and a high level of 17β-HSD1 [58]. Thus, the T-47D cell line offers the opportunity to delineate the action of a 17β-HSD1 inhibitor on two pathways producing an estrogenic effect. Advantageously, we had the option to choose to stimulate the cell with either DHEA (to obtain 5-diol) or E1 (to obtain E2) and to observe the corresponding action of a 17β-HSD1 inhibitor on those two transformations. Otherwise, MCF-7 cells are known to express a lower level of 17β-HSD1 and a higher expression of aromatase than T-47D cells, suggesting a dominance of 4-dione to the E1 to E2 pathway in estrogen formation [50,58]. This pathway preference was highlighted by the capacity of an aromatase inhibitor to block 4-dione-stimulated MCF-7 cell proliferation [59]. Thus, the use of MCF-7 cells would be non-conclusive to assign a 17β-HSD1 contribution in 5-diol formation. ZR-75-1 cells show a very weak capacity to transform E1 to E2, limiting their utility to determine the role of a 17β-HSD1 inhibitor in this transformation [50]. Consequently, we limited our study to T-47D cells, considering this ER(+) cell line as the sole line with the required enzyme (17β-HSD1/aromatase) profile expression toward our main study objective. In a personalized medicine perspective, T-47D cells are also representative of breast cancer patients overexpressing 17β-HSD1 and with a low level of aromatase.

In the first part of the present study using T-47D cells, we showed 5-diol to be the major estrogen, with a very weak formation of E2. We also showed that 17β-HSD1 was responsible for its formation from DHEA, an abundant steroid in women. This transformation was blocked in T-47D cells by the use of the targeted-covalent 17β-HSD1 inhibitor PBRM. More importantly, in mice, the PBRM inhibitor was able to reverse the growth of ER(+) tumors stimulated by DHEA in a breast cancer model (cell-derived T-47D xenografts).

In a preliminary study, we showed the ability of PBRM (15 mg/kg, SC) to block the growth of ER(+) tumors (T-47D xenografts) stimulated by E1, the precursor of E2. In the second part of the present study, we reproduced this result and demonstrated its effectiveness when administered orally (PO), a clear asset toward drug development. A dose–response experiment revealed the effectiveness of PBRM at doses of 30 and 15 mg/kg PO, but also at 5 mg/kg PO. At this dose, however, more time was required to block tumor growth. The irreversible nature of the PBRM inhibitor is its main characteristic, which differentiates it from other known 17β-HSD1 reversible inhibitors [39]. During a second study for the transformation of E1 into E2 (T-47D xenografts), we reduced the frequency of administration of the inhibitor for the same dose (15 mg/kg) and were able to demonstrate the advantage of an irreversible inhibitor such as PBRM. Indeed, PBRM was similarly effective when administered three or six times per week and remained very effective when administered only once a week. This behavior is explained by the formation of a covalent bond between the enzyme (His-221) and the bromoethyl side chain of PBRM [45]. This result obtained in an animal model of breast cancer with the targeted-covalent inhibitor PBRM undeniably demonstrates its advantage over a reversible inhibitor that must be administered more frequently (daily).

The first-generation irreversible inhibitor class had low selectivity of action, which greatly reduced the therapeutic potential. Worse yet, this class of inhibitor is responsible for a lingering feeling still present today in the scientific and pharmaceutical communities that irreversible inhibitors cause unwanted side effects due to their lack of selectivity. Fortunately, the new generations of irreversible inhibitors, now called targeted-covalent inhibitors [54,55,56], have a high selectivity of action, due to their design, where covalent binding is favored by the active site amino acid environment of the biological target. The PBRM inhibitor is part of this new generation of selective irreversible inhibitors. In fact, no side effects or indications of lack of selectivity on the part of PBRM were detected in the experiments (xenografts) performed in mice, which lasted for approximately one month. In addition, a study to determine the maximum tolerable dose in mice did not raise any problems other than the appearance of diarrhea due to the use of sunflower oil as the main constituent of the delivery vehicle, and only at the highest dose of 1350 mg/kg [60]. PBRM therefore stands out from other known 17β-HSD1 inhibitors which are reversible in nature [39].

For diseases where estrogens have a detrimental effect, such as breast cancer and endometriosis, we demonstrated the importance of 5-diol as another estrogen source. Produced directly from DHEA, an abundant C19-steroid, 5-diol could be an alternative to classically-produced E2 (DHEA to 4-dione to E1 to E2) before menopause, but it is also likely to play a non-negligible role for cancer cells whose production of E2 is deficient. Keep in mind that an aromatase inhibitor (AI) will not prevent the formation of 5-diol, unlike an inhibitor of 17β-HSD1. In a personalized medicine optic, the expression profile of 17β-HSD1/aromatase in a patient should be a crucial factor to consider when choosing the appropriate inhibitor for treatment. Indeed, Sasano et al. [61] showed a high degree of intratumor variability of these enzyme expressions between patients. In the case of T-47D, this breast cancer cell type represents a particular case of a patient expressing 17β-HSD1 almost exclusively with a very low expression of aromatase, and who could be treated following a 17β-HSD1 inhibitor monotherapy. Otherwise, other 17β-HSDs could potentially contribute to estrogen biosynthesis with the ability to transform E1 into E2, such as 17β-HSD7 [62], 17β-HSD12 [63], 17β-HSD5 (AKR1C3) [64] and DHRS11 (dehydrogenase/reductase SDR family member 11) [65], but their contribution to the production of E2 in the different ER(+) cancer cell lines and tumors has yet to be clarified.

## 4. Materials and Methods

### 4.1. Inhibitors

PBRM, a targeted-covalent steroidal inhibitor of 17β-HSD1, was synthesized, as previously reported [66]. Compound **21** [57], a reversible steroidal inhibitor of 17β-HSD1 used in our study, was synthesized in our laboratory using published procedures.

### 4.2. In Vitro Studies

#### 4.2.1. Cell Culture

Human breast cancer T-47D cells were obtained from the American Type Culture Collection (ATCC) and maintained in a 175 cm^2^ culture flask at 37 °C in a humidified atmosphere at 5% CO_2_. Cells were grown in RPMI medium supplemented with 10% (v/v) fetal bovine serum (FBS), L-glutamine (2 nM), penicillin (100 IU/mL), streptomycin (100 µg/mL) and 17β-estradiol (1 nM). T-47D cells were used for the DHEA metabolism assays, 17β-HDSD1 inhibition assays and xenograft experiments.

#### 4.2.2. Metabolism of DHEA in T-47D Cells

T-47D cells (3000, 6000, 12,000 or 24,000) were plated in 24-well culture. After incubation for 1 day, 10 μL of a 50 μM [^14^C]-DHEA solution (Perkin Elmer, Boston, MA, USA) in culture medium (990 μL) were added. After 1, 2, 3, 6 or 8 days, the culture medium was recovered from the wells and steroids were extracted with diethyl ether. After evaporating the organic phase to dryness with a nitrogen stream, the residue was dissolved in dichloromethane, dropped on silica gel 60F254 thin-layer chromatography (TLC) plates (Milipore-Sigma, Oakville, ON, Canada) and eluted with a mixture of toluene:acetone (4:1), [^14^C]-labeled steroids (DHEA, 5-diol, 4-dione, T, DHT, E1, E2, ADT and 3β-diol) were identified by comparison with reference steroids and quantified using a Storm 860 System (Molecular Dynamics, Sunnyvale, CA, USA). The relative abundance of each steroid was calculated and expressed in percentage. In one experiment, steroids (DHEA, 5-diol, 4-dione, DHT and E2) were quantified by gas chromatography–mass spectrometry (GC-MS/MS) analysis according to an established procedure developed at the CHU de Québec (CHUL)-Research Center and previously published [65].

#### 4.2.3. 17β-HSD1 Inhibition Assays

T-47D cells (24,000) were seeded in a 24-well plate in 980 µL of medium supplemented with insulin (50 ng/mL) and 5% dextran-coated charcoal-treated FBS, which was used rather than untreated 10% FBS, to remove the remaining steroid hormones. A stock solution of PBRM inhibitor was previously prepared in DMSO and diluted with culture medium to achieve appropriate concentrations prior to use. After 24 h of incubation, 10 µL of the diluted solution of PBRM were added to the cells to obtain the appropriate final concentration. The final concentration of DMSO in the well was adjusted to 0.1%. Additionally, 10 µL of a solution of [^14^C]-DHEA (Perkin Elmer, Boston, MA, USA) were added to obtain a final concentration of 0.5 μM. Cells were incubated for 8 days and each inhibitor was assessed in triplicate. After incubation, the culture medium was removed and labeled steroids (5-diol and DHEA) were extracted with diethyl ether. The organic phases were combined and evaporated to dryness with nitrogen. Residues were dissolved in dichloromethane and dropped on silica gel 60F254 TLC and eluted with toluene⁄acetone (4:1) as solvent system. Substrate [^14^C]-DHEA and metabolite [^14^C]-5-diol were identified by comparison with reference steroids (DHEA and 5-diol) and quantified using the Storm 860 system (Molecular Dynamics, Sunnyvale, CA, USA). The percentage of transformation and the percentage of inhibition were calculated, as previously reported [43].

### 4.3. In Vivo Studies (T-47D Xenografts in Nude Mice)

#### 4.3.1. Animals for Xenografts

All experiments were conducted in an animal facility approved by the Canadian Council on Animal Care (CCAC) and the Association for Assessment and Accreditation of Laboratory Animal Care, and the protocols were approved by the Institutional Animal Ethics Committee (Université Laval, Québec, QC, Canada). All mice were acclimatized to the environmental conditions (temperature, 22 ± 3 °C; humidity, 50 ± 20%; 12 h light/dark cycles, lights on at 07:15 h) for at least 3–5 days before starting the xenograft experiments. Homozygous female nu/nu Br athymic mice (28–42 days old, 21–29 g) from Charles River, Inc. (Saint-Constant, QC, Canada) were housed in vinyl cages (4–5 mice/cage) equipped with air lids, kept in laminar airflow hoods and maintained under pathogen-limiting conditions. Cages, bedding, water and food (Teklad global 18% protein #2018SX, Envigo, Madison, WI, USA) were autoclaved before use. After the acclimation period, bilateral ovariectomy was performed under isoflurane-induced anesthesia, and mice were treated 6 times per week with DHEA (1 or 3 mg in 0.05 mL of propylene glycol:EtOH (92:8)/mouse) or E1 (0.1 μg/0.05 mL in propylene glycol:EtOH (92:8)/mouse). Three days after the surgery, 10^7^ T-47D cells were inoculated SC in 0.05 mL of RPMI medium containing 92% Matrigel in both flanks of each mouse through a 2.5 cm-long 22-gauge needle. After 6 weeks of stimulation with DHEA or 3 weeks of stimulation with E1, randomization and treatment were started. One day prior to initiation of treatment, all mice bearing tumors were randomly assigned to all groups (with respect to tumor size). All treatment solutions were prepared one day prior to initiation of treatment, stored at 4 °C and kept under constant agitation. The tumor size was measured two times a week with a caliper. Two perpendicular diameters (L and W) were recorded and the tumor area (mm^2^) was calculated using the formula L/2 × W/2 × π. The area measured on the first day of treatment was taken as 100%.

#### 4.3.2. Stimulation of T-47D Tumor Growth by DHEA in Nude OVX Mice

Mice were divided into Group 1 (OVX-CTL, 2 mice, 4 tumors), Group 2 (OVX + DHEA (1 mg/day), 2 mice, 4 tumors) and Group 3 (OVX + DHEA (3 mg/day), 2 mice, 4 tumors). DHEA was given 6 times per week by SC injection in 0.05 mL of propylene glycol:EtOH (92:8) for 32 days and all mice in the OVX-CTL group received only the vehicle.

#### 4.3.3. Inhibition of DHEA-Stimulated T-47D Tumor Growth in Nude OVX Mice

Mice were divided into Group 1 (OVX + DHEA (3 mg/day), 3 mice, 6 tumors) and Group 2 (OVX + DHEA (3 mg/day) + PBRM (15 mg/kg) PO, 3 mice, 6 tumors). All treatments were given 6 times per week for 30 days. DHEA was given by SC injection in 0.05 mL of propylene glycol:EtOH (92:8) and PBRM was given orally (PO) by gavage administration in 0.1 mL of sunflower oil:EtOH (92:8). At the end of the protocol, and 3 h after the last treatment, mice were weighed, anesthetized with isoflurane, and killed by exsanguination (cardiac puncture).

#### 4.3.4. Inhibition of E1-Stimulated T-47D Tumor Growth in Nude OVX Mice (Increased Dose of PBRM)

Mice were divided into Group 1 (OVX-CTL, 7 mice, 14 tumors), Group 2 (OVX + E1 (0.1 μg/day), 7 mice, 14 tumors), Group 3 (OVX + E1 (0.1 μg/day) + PBRM (5 mg/kg) PO, 8 mice, 16 tumors), Group 4 (OVX + E1 (0.1 μg/day + PBRM (15 mg/kg) PO, 8 mice, 16 tumors), Group 5 (OVX + E1 (0.1 μg/day) + PBRM (30 mg/kg) PO, 8 mice, 16 tumors) and Group 6 (OVX + E1 (0.1 μg/day) + PBRM (15 mg/kg) SC, 8 mice, 16 tumors). All treatments were given 6 times per week, and all mice in the OVX-CTL group received only the vehicle for 30 days. E1 was given by SC injection in 0.05 mL of propylene glycol:EtOH (92:8), PBRM was given either by SC injection in 0.1 mL of propylene glycol:EtOH (92:8) or by PO administration in 0.1 mL of sunflower oil:EtOH (92:8). At the end of the protocol, and 3 h after the last treatment, mice were weighed, anesthetized with isoflurane and killed by exsanguination (cardiac puncture). Blood and tumors were removed and immediately frozen at −80 °C. Uterus, kidney and liver were removed and weighed.

#### 4.3.5. Inhibition of E1-Stimulated T-47D Tumor Growth in Nude OVX Mice (Frequency of PBRM Administration)

Mice were divided into Group 1 (OVX-CTL, 8 mice, 16 tumors), Group 2 (OVX + E1 (0.1 μg/day), 8 mice, 15 tumors), Group 3 (OVX + E1 (0.1 μg/day) + PBRM (15 mg/kg) PO, 1 time/week, 8 mice, 16 tumors), Group 4 (OVX + E1 (0.1 μg/day) + PBRM (15 mg/kg) PO, 3 times/week, 8 mice, 16 tumors), Group 5 (OVX + E1 (0.1 μg/day) + PBRM (15 mg/kg) PO, 6 times/week, 8 mice, 16 tumors) and Group 6 (OVX + E1 (0.1 μg/day) + compound **21** [57] (15 mg/kg) PO, 1 time/week, 7 mice, 14 tumors). All treatments of E1 were given 6 times per week and all mice in the CTL group received only the vehicle for 28 days. E1 was given by SC injection in 0.05 mL of propylene glycol:EtOH (92:8), PBRM and compound **21** [57] was given by PO administration in 0.1 mL of sunflower oil:EtOH (92:8). At the end of the protocol, and 3 h after the last treatment, mice were weighed, anesthetized with isoflurane and killed by exsanguination (cardiac puncture). Uterus, kidney and liver were removed and weighed.

### 4.4. Dosage of Steroids and PBRM

The concentration of steroids (DHEA, 5-diol, 4-dione, DHT and E2) were determined by GC-MS/MS analysis according to an established procedure developed at the CHU de Québec (CHUL)-Research Center and previously published [67]. The concentration of PBRM in blood (serum) was determined by LC-MS/MS analysis, as previously reported [42]. The same LC-MS/MS methodology was also used to determine the concentration of PBRM in tumors. However, before PBRM dosage, the tumors from the same group were pooled and processed, as previously reported for different tissues [68].

### 4.5. Statistical Analysis

Statistical significance was determined according to Duncan’s multiple-range test [69]. *p* values which were less than 0.05 were considered as statistically significant.

## 5. Conclusions

Our results show the importance of 17β-HSD1 in breast cancer T-47D ER(+) cells for the formation of 5-diol, an estrogen produced by another route than the one producing E2. We also showed the ability of the targeted-covalent inhibitor PBRM to effectively inhibit both the transformation of E1 to E2 and that of DHEA to 5-diol, therefore blocking the biosynthesis of two estrogens that can stimulate the growth of estrogen-dependent tumors in a breast cancer model (cell-derived T-47D xenografts). It should also be noted that the irreversibility of this second-generation selective inhibitor made it possible to limit the frequency of administration of this orally active 17β-HSD1 inhibitor to once a week, a clear asset over reversible inhibitors. Having not demonstrated any apparent toxicity in studies in mice (xenografts, maximum tolerable dose, etc.), PBRM as a targeted-covalent inhibitor of 17β-HSD1 is in a good position to progress toward clinical studies.

## Figures and Tables

**Table 1:** Effect of enzyme inhibitors on DHEA metabolism in T-47D breast cancer cells ^1^.

Steroids	LLOQ (ng/mL)	DHEA (ng/mL)	DHEA (%)	DHEA + PBRM (ng/mL)	DHEA + PBRM (%)	DHEA + LET (ng/mL)	DHEA + LET (%)	DHEA + PBRM + LET (ng/mL)	DHEA + PBRM + LET (%)
DHEA	0.10	905	77.9	967	84.7	916	76.9	1051	85.0
5-diol	0.05	244	21.0	159	13.9	261	21.9	169	13.7
4-dione	0.05	12.1	1.04	15.7	1.4	14.0	1.2	16.4	1.32
DHT	0.01	0.34	0.03	0.23	Tr	0.23	Tr	0.24	0.02
E2	0.005	0.18	0.01	0.20	Tr	0.22	Tr	0.22	0.02

^1^ Intact T-47D cells were incubated for eight days in the presence of DHEA, PBRM (1 μM) and LET (1 μM). Steroids were extracted and quantified by GC-MS/MS. PBRM is a steroidal inhibitor of 17β-HSD1 and LET (letrozole) is a nonsteroidal inhibitor of aromatase. Tr, trace of detected steroid; LLOQ, lower limit of quantification; DHEA, dehydroepiandrosterone; 5-diol, 5-androstene-3,17-diol; 4-dione, 4-androstene-3,17-dione; DHT, dihydrotestosterone; E2, estradiol.

## Data Availability

Data is contained within the article or supplementary material.

## Supplementary Materials

The following are available online at https://www.mdpi.com/article/10.3390/cancers13081841/s1, Figures S1–S6 and Tables S1–S7: Transformation of DHEA and 4-dione in T-47D cells (different cell numbers and times).
