# Supplementary material for: A Targeted-Covalent Inhibitor of 17β-HSD1 Blocks Two Estrogen-Biosynthesis Pathways: In Vitro (Metabolism) and In Vivo (Xenograft) Studies in T-47D Breast Cancer Models
Source: Cancers (Basel). 2021 Apr 13;13(8):1841. doi: 10.3390/cancers13081841 (PMC8069897; doi:10.3390/cancers13081841)
Supplement: Supplementary file 1 [file cancers-13-01841-s001.pdf]

Supplementary Materials

Transformation of DHEA and 4-dione in T-47D cells

(see the manuscript section 4,2,2 in Materials and Methods for the experimental details)

Detection and quantification of steroids by TLC (thin-layer chromatography) and radioactivity measurement or GC-MS/MS (gas chromatography tandem mass spectrometry)

1) [^{14}C]-DHEA; 24 000 cells; 3, 6 and 8 days; TLC (Figure S1 and Table S1)
2) [^{14}C]-4-dione; 24 000 cells; 3, 6 and 8 days; TLC (Figure S2 and Table S2)
3) [^{14}C]-DHEA; 6000, 12 000 and 24 000 cells; 6 days; TLC (Figure S3 and Table S3)
4) [^{14}C]-4-dione; 6000, 12 000 and 24 000 cells; 6 days; TLC (Figure S4 and Table S4)
5) [^{14}C]-4-dione; 6000 cells; 1, 2, 3 and 6 days; TLC (Figure S5 and Table S5)
6) [^{14}C]-4-dione; 3000 cells; 1, 2, 3 and 6 days; TLC (Figure S6 and Table S6)
7) Proportion of steroids detected by GC-MS/MS from two incubations with DHEA (Table S7)

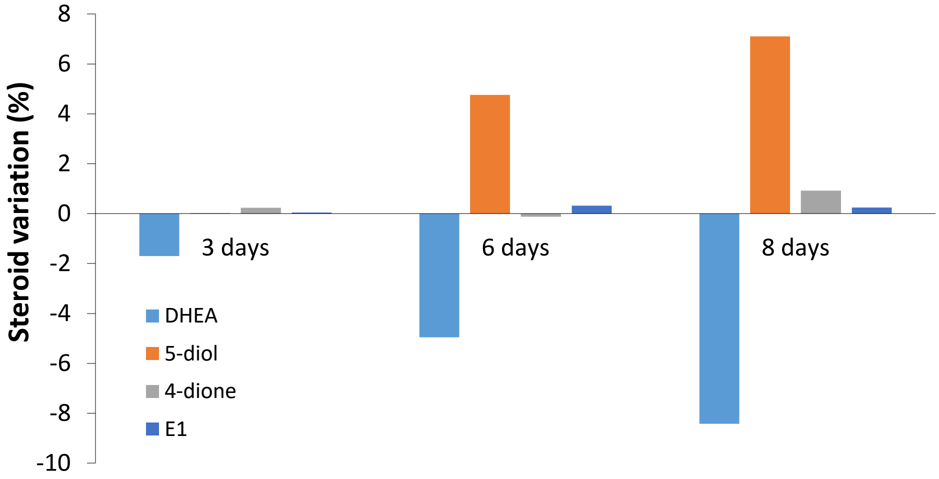

Figure S1. [14C]-DHEA; 24 000 cells; 3, 6 and 8 days; TLC (Figure S1 and Table S1).

Table S1. [14C]-DHEA; 24 000 cells; 3, 6 and 8 days; TLC.

24000 cells	Steroid variation (%)		
	3 days	6 days	8 days
DHEA	-1.7	-4.96	-8.42
5-diol	0.02	4.76	7.11
4-dione	0.23	-0.12	0.92
E1	0.04	0.32	0.25

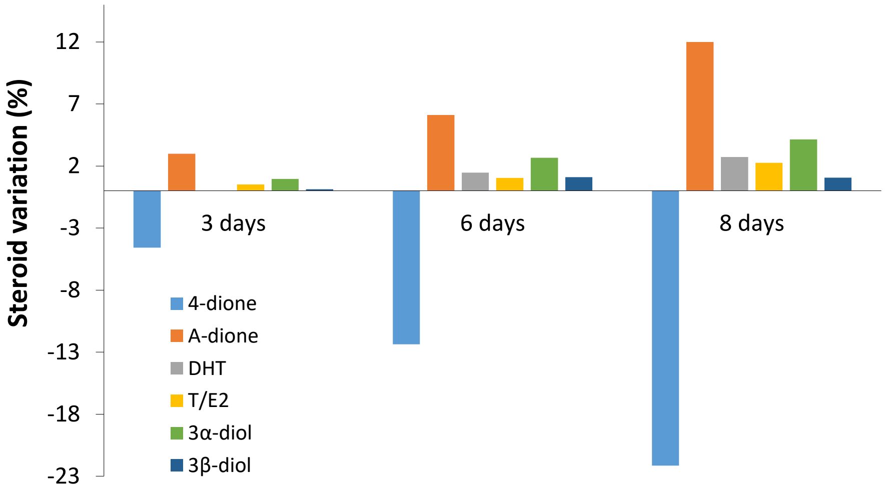

Figure S2. [14C]-4-dione; 24 000 cells; 3, 6 and 8 days; TLC (Figure S2 and Table S2).

Table S2. [14C]-4-dione; 24 000 cells; 3, 6 and 8 days; TLC.

24000 cells	Steroid variation (%)		
	3 days	6 days	8 days
4-dione	-4.58	-12.38	-22.15
A-dione	2.98	6.11	11.99
DHT	0	1.47	2.72
T/E2	0.52	1.03	2.26
3α-diol	0.95	2.67	4.15
3β-diol	0.12	1.1	1.05

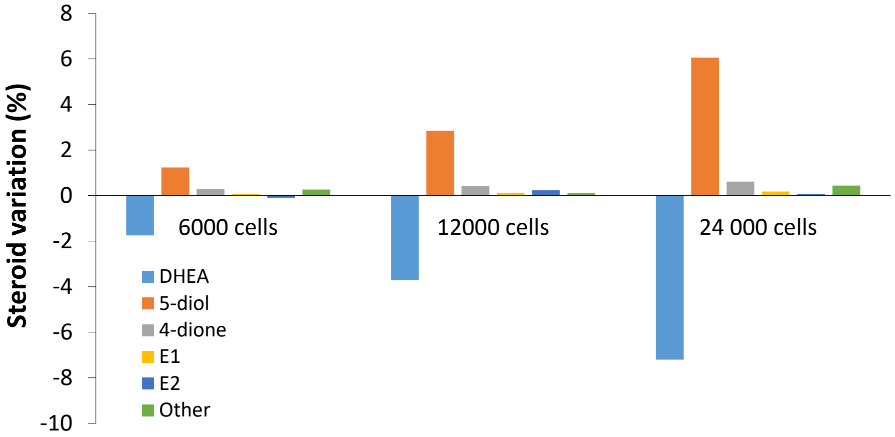

Figure S3. [14C]-DHEA; 6000, 12 000 and 24 000 cells; 6 days; TLC (Figure S3 and Table S3).

Table S3. [14C]-DHEA; 6000, 12 000 and 24 000 cells; 6 days; TLC.

	Steroid variation (%)		
	**6000 cells**	**12000 cells**	**24 000 cells**
**DHEA**	-1.75	-3.71	-7.21
**5-diol**	1.23	2.85	6.06
**4-dione**	0.29	0.42	0.61
**E1**	0.07	0.12	0.18
**E2**	-0.09	0.23	0.07
**Other**	0.27	0.1	0.44

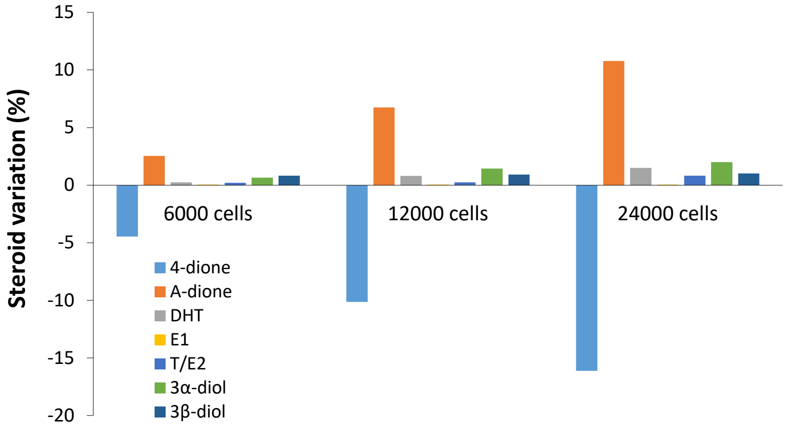

Figure S4. [C]-4-dione; 6000, 12 000 and 24 000 cells; 6 days; TLC (Figure S4 and Table S4).

Table S4. [C]-4-dione; 6000, 12 000 and 24 000 cells; 6 days; TLC.

	Steroid variation (%)		
	**6000 cells**	**12000 cells**	**24000 cells**
**4-dione**	-4.47	-10.14	-16.13
**A-dione**	2.54	6.75	10.79
**DHT**	0.25	0.8	1.49
**E1**	0.05	0.05	0.05
**T/E2**	0.21	0.24	0.82
**3α-diol**	0.64	1.43	2
**3β-diol**	0.82	0.91	1.02

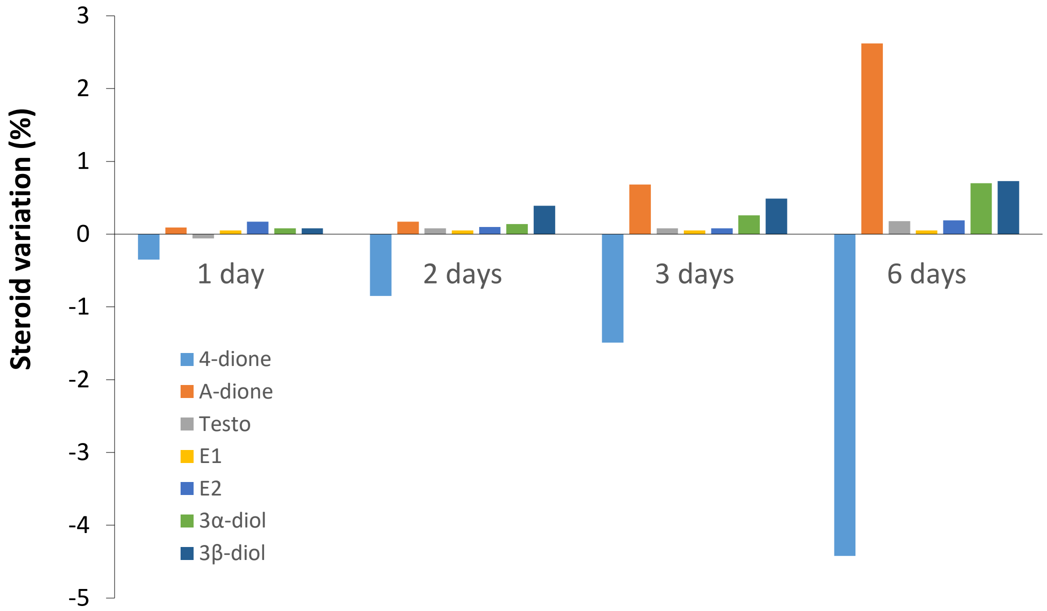

Figure S5. [14C]-4-dione; 6000 cells; 1, 2, 3 and 6 days; TLC (Figure S5 and Table S5).

Table S5. [14C]-4-dione; 6000 cells; 1, 2, 3 and 6 days; TLC.

	Steroid variation (%)			
	**1 day**	**2 days**	**3 days**	**6 days**
**4-dione**	-0.35	-0.85	-1.49	-4.42
**A-dione**	0.09	0.17	0.68	2.62
**Testo**	-0.06	0.08	0.08	0.18
**E1**	0.05	0.05	0.05	0.05
**E2**	0.17	0.1	0.08	0.19
**3α-diol**	0.08	0.14	0.26	0.7
**3β-diol**	0.08	0.39	0.49	0.73

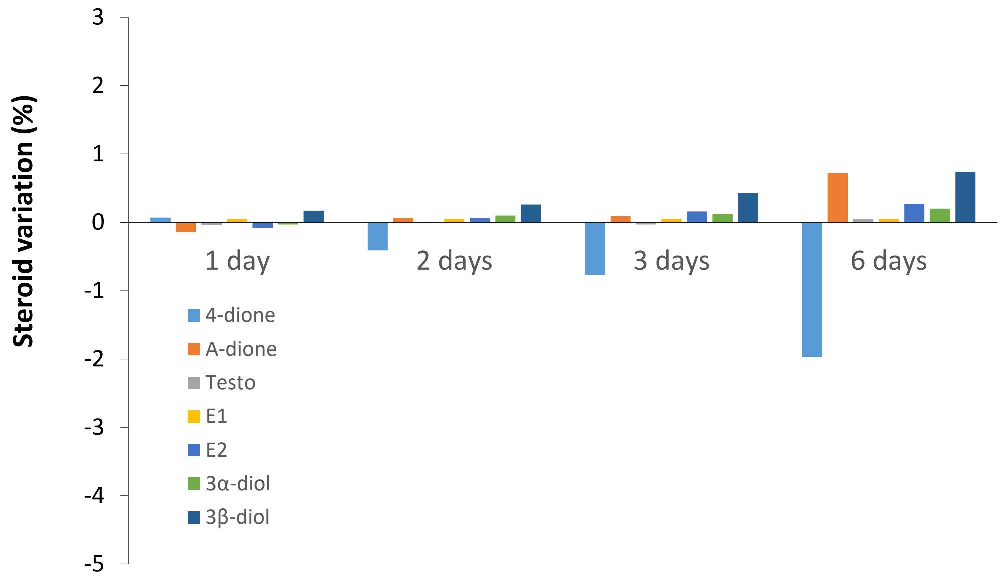

Figure S6. [14C]-4-dione; 3000 cells; 1, 2, 3 and 6 days; TLC (Figure S6 and Table S6).

Table S6. [14C]-4-dione; 3000 cells; 1, 2, 3 and 6 days; TLC

	Steroid variation (%)			
	**1 day**	**2 days**	**3 days**	**6 days**
**4-dione**	0.07	-0.41	-0.77	-1.97
**A-dione**	-0.14	0.06	0.09	0.72
**Testo**	-0.04	-0.01	-0.03	0.05
**E1**	0.05	0.05	0.05	0.05
**E2**	-0.08	0.06	0.16	0.27
**3α-diol**	-0.03	0.1	0.12	0.2
**3β-diol**	0.17	0.26	0.43	0.74

Table S7. Proportion of steroids detected by GC-MS/MS from two incubations (A and B) with DHEA. A and B: DHEA incubated 8 days in 24 000 T-47D cells.

**Steroids**	**LLOQ (ng/mL)**	**A (ng/mL)**	**A (%)**	**B * (ng/mL)**	**B * (%)**
DHEA	0.10	1130	79.6	905	77.9
5-diol	0.05	236	16.6	244	21.0
4-dione	0.05	27	1.9	12.1	1.04
DHT	0.01	0.3	0.02	0.34	0.03
T	0.03	1.9	0.13	NA	NA
3β-diol	0.01	0.1	0.007	NA	NA
ADT	0.05	0.7	0.05	NA	NA
E1	0.005	23	1.6	NA	NA
E2	0.005	0.9	0.06	0.18	0.01

(*) Data from Table 1 (DHEA alone). LLOQ: Lower limit of quantification; DHEA: dehydroepiandrosterone; 5-diol: 5-androstene-3,17-dione; 4-dione: 4-androstene-3,17-dione; DHT: dihydrotestosterone; T: testosterone; 3β-diol: 5α-androstan-3β,17β-diol; ADT: androsterone; E1: estrone; E2: estradiol. NA: not available.
